# Glioma and temozolomide induced alterations in gut microbiome

**DOI:** 10.1038/s41598-020-77919-w

**Published:** 2020-12-03

**Authors:** Anthony Patrizz, Antonio Dono, Soheil Zorofchian, Gabriella Hines, Takeshi Takayasu, Nuruddin Husein, Yoshihiro Otani, Octavio Arevalo, H. Alex Choi, Jude Savarraj, Nitin Tandon, Bhanu P. Ganesh, Balveen Kaur, Louise D. McCullough, Leomar Y. Ballester, Yoshua Esquenazi

**Affiliations:** 1 Vivian L. Smith Department of Neurosurgery, The University of Texas Health Science Center at Houston, McGovern Medical School, Houston, TX USA; 2 Department of Pathology and Laboratory Medicine, The University of Texas Health Science Center at Houston, McGovern Medical School, Houston, TX USA; 3 Department of Diagnostic and Interventional Imaging, The University of Texas Health Science Center at Houston, McGovern Medical School, Houston, TX USA; 4 Department of Neurology, The University of Texas Health Science Center At Houston, McGovern Medical School, Houston, TX USA; 5 Center for Precision Health, The University of Texas Health Science Center At Houston, McGovern Medical School, Houston, TX USA; 6 Memorial Hermann Hospital-TMC, Houston, TX USA; 7 Vivian L. Smith Department of Neurosurgery and Center for Precision Health, The University of Texas Health Science Center at Houston – McGovern Medical School, 6400 Fannin Street, Suite # 2800, Houston, TX 77030 USA; 8 Department of Pathology & Laboratory Medicine and Department of Neurosurgery, The University of Texas Health Science Center at Houston – McGovern Medical School, 6431 Fannin Street, MSB 2.136, Houston, TX 77030 USA

**Keywords:** CNS cancer, Cancer in the nervous system

## Abstract

The gut microbiome is fundamental in neurogenesis processes. Alterations in microbial constituents promote inflammation and immunosuppression. Recently, in immune-oncology, specific microbial taxa have been described to enhance the effects of therapeutic modalities. However, the effects of microbial dysbiosis on glioma are still unknown. The aim of this study was to explore the effects of glioma development and Temozolomide (TMZ) on fecal microbiome in mice and humans. C57BL/6 mice were implanted with GL261/Sham and given TMZ/Saline. Fecal samples were collected longitudinally and analyzed by 16S rRNA sequencing. Fecal samples were collected from healthy controls as well as glioma patients at diagnosis, before and after chemoradiation. Compared to healthy controls, mice and glioma patients demonstrated significant differences in beta diversity, *Firmicutes/Bacteroides* (F/B) ratio, and increase of *Verrucomicrobia* phylum and *Akkermansia* genus. These changes were not observed following TMZ in mice. TMZ treatment in the non-tumor bearing mouse-model diminished the F/B ratio, increase *Muribaculaceae* family and decrease *Ruminococcaceae* family. Nevertheless, there were no changes in *Verrucomicrobia*/*Akkermansia*. Glioma development leads to gut dysbiosis in a mouse-model, which was not observed in the setting of TMZ. These findings seem translational to humans and warrant further study.

## Introduction

Glioma is the most common primary malignant tumor of the Central Nervous System (CNS), with 50% of patients presenting with the most aggressive type, glioblastoma (GBM)^1^. In spite of recent advances in chemoradiation and surgical approaches, the median survival continues to be uniformly poor^2,3^. Despite progress in basic and clinical research, the overall survival (OS) of GBM has not significantly increased for the past decades^3^. The rapid proliferation, infiltrating nature, molecular heterogeneity, and challenges to deliver drugs across the blood–brain barrier have limited the effectiveness of therapies^4^. Development of new therapeutic strategies through the study of molecular and genomic characteristics, tumor microenvironment, and tumor-host interactions has become of great interest in the need to develop new potential therapies.

The human gastrointestinal microbiome is composed of several microorganisms with diverse natures and functions that are dependent on genetic and environmental factors^5–8^. In recent years, a growing body of evidence supports the role of the gut microbiome (GM) in human physiology and biological activity under homeostatic and pathological conditions^6,9^. Evidence now suggests that some microbes may confer susceptibility to certain cancers and may influence response to therapies^10–13^. Gut dysbiosis, a disequilibrium in the bacterial ecosystem, leads to overrepresentation of some bacteria and favors chronic inflammation and immunosuppression^14^, as recently described in several CNS diseases, including stroke, Parkinson’s disease, amyotrophic lateral sclerosis (ALS), and multiple sclerosis (MS)^5,15–19^. However, the relationship between the gut-brain axis, glioma development, and how the GM responds and interacts with Temozolomide (TMZ) has not been investigated. The goals of this study were (1) to explore the GM changes following glioma growth and after oral TMZ in a glioma mouse-model and (2) to identify glioma induced GM changes and the effects of TMZ in a prospective glioma patient cohort.

## Results

### Glioma leads to dysbiosis in the gut microbiome in mice

**Figure 1:**
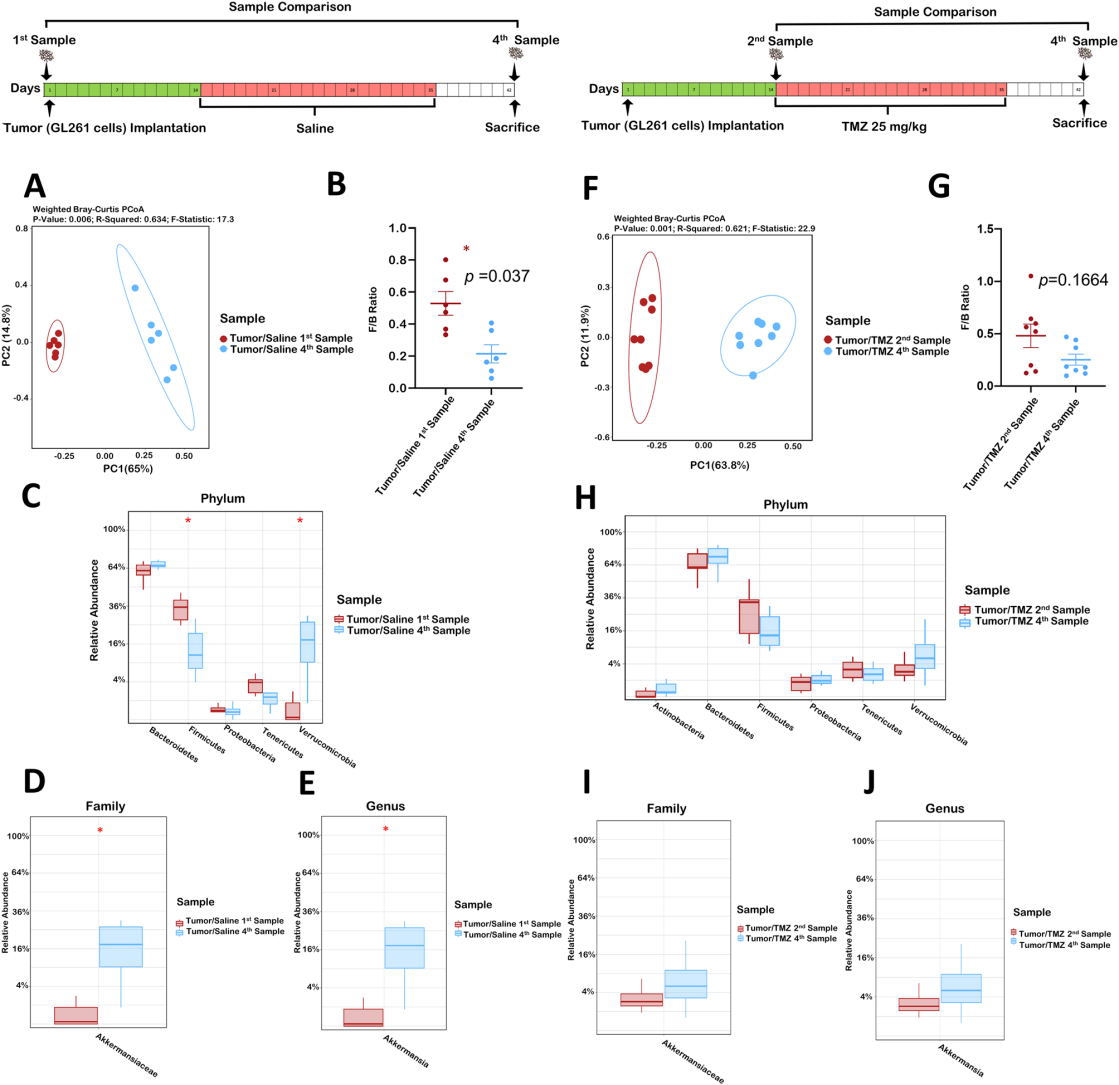
(**A**–**E**) Glioma induces dysbiosis in the mouse gut microbiome. Comparison of Tumor/Saline 1st and 4th samples (n = 6). (**A**) Beta diversity Weighted Bray–Curtis PCoA. (**B**) *Firmicutes* to *Bacteroides* ratio. (**C**) Relative abundance taxa summary at the Phylum level. (**D**) Relative abundance of *Akkermansiaceae* at the family level. (**E**) Relative abundance of *Akkermansia* at the genus level. (**F–J**) *Temozolomide reshapes the Glioma-induced Dysbiosis.* Comparison of Tumor/TMZ 2nd and 4th samples (n = 8). (**F**) Beta diversity Weighted Bray–Curtis PCoA. (**G**) *Firmicutes* to *Bacteroides* ratio. (**H**) Relative abundance taxa summary at the Phylum level. (**I**) Relative abundance of *Akkermansiaceae* at the family level. (**J**) Relative abundance of *Akkermansia* at the genus level.

First, we performed a comparison of samples before tumor implantation (1st sample-baseline) vs. sacrifice (4th sample) to evaluate if glioma growth changes the GM. This comparison revealed that the operational taxonomic units (OTUs) were significantly different (*p* = 0.0049) while the Shannon index showed similar bacterial community richness and diversity (*p* = 0.065, Supplementary Fig. S1A). Further assessment using the Principal coordinate analysis (PCoA) of weighted pairwise Bray–Curtis distances demonstrated differential clustering patterns (*p* = 0.006), Fig. 1A. There was a significant decrease in the *Firmicutes* to *Bacteroides* (F/B) ratio suggesting dysbiosis following tumor-growth (*p* = 0.037, Fig. 1B). The relative abundance after tumor development demonstrated decreased *Firmicutes* (*p* = 0.03) and increased *Verrucomicrobia* phyla (*p* = 0.03, Fig. 1C).

*Akkermansiaceae* family*,* the dominant family in the phylum *Verrucomicrobia*^20^ and its genus, *Akkermansia* were evaluated, demonstrating an increased relative abundance between the 1st and 4th sample in tumor-bearing mice (*p* = 0.03 and 0.035, respectively, Fig. 1D,E).

To confirm that the observed changes were secondary to tumor growth rather than weight loss, we performed a similar analysis between the 1st and 3rd samples (collected prior to bodyweight loss). Results demonstrated similar changes in alpha diversity, F/B ratio, PCoA, *Verrucomicrobia*, *Akkermansiaceae* and *Akkermansia* (Supplementary Fig. S2). Furthermore, the 1st and 4th samples comparison in the Sham/Saline group did not display changes in *Verrucomicrobia*, *Akkermansiaceae*, or *Akkermansia* (Supplementary Fig. S3).

Since GBM typically occurs within the 5–7th decade^21^, and aging alters the microbiome composition^22^; we implanted GL261 cells into 18–20-month-old mice. Alpha diversity indices were comparable to young mice at baseline (Supplementary Fig. S4A). Consistent with previous findings^22^, the F/B ratio in aged mice was increased at baseline, suggestive of underlying dysbiosis (Supplementary Fig. S4B). PCoA exhibited separate clusters in weighted Bray–Curtis distances between young and aged mice (Supplementary Fig. S4C). The aged microbiome differed from young in the relative abundance of *Bacteroidetes, Firmicutes, Tenericutes,* and *Actinobacteria* phyla (Supplementary Fig. S4D). However, the relative abundance of *Verrucomicrobia*, *Akkermansiaceae*, and *Akkermansia* remained unchanged with aging (Supplementary Fig. S4D–F). Following tumor implantation, there was a similar pattern of microbiome alteration in aged and young mice (Supplementary Fig. S5). Overall our results demonstrated that glioma development leads to a GM dysbiosis in mice.

### Oral temozolomide prevented the glioma-induced dysbiosis

Then, we investigated the TMZ (oral chemotherapy) effect on glioma-induced dysbiosis, which is the standard of care chemotherapy for GBM. Mice were gavaged with TMZ (25 mg/kg) for three-weeks after tumor implantation. Stool analysis was performed between the 2nd (before treatment) and 4th samples (before sacrifice). Our results demonstrated no differences in OTUs or Shannon index (Supplementary Fig. S1B). Although the fecal samples (FS) in the 2nd and 4th samples in the Tumor/TMZ group clustered separately (*p* = 0.001, Fig. 1F) there was no difference in the F/B ratio (*p* = 0.17, Fig. 1G). In addition, our results did not demonstrate changes at the *Firmicutes* and *Verrucomicrobia* phyla or *Akkermansiaceae* family and *Akkermansia* genus (Fig. 1H–J). Similar results were observed in aged mice (Supplementary Fig. S5). Collectively, our results in mice, suggest that the glioma-induced microbiome changes were not observed in the TMZ setting.

### Effect of oral temozolomide on non-tumor bearing mice microbiome

**Figure 2:**
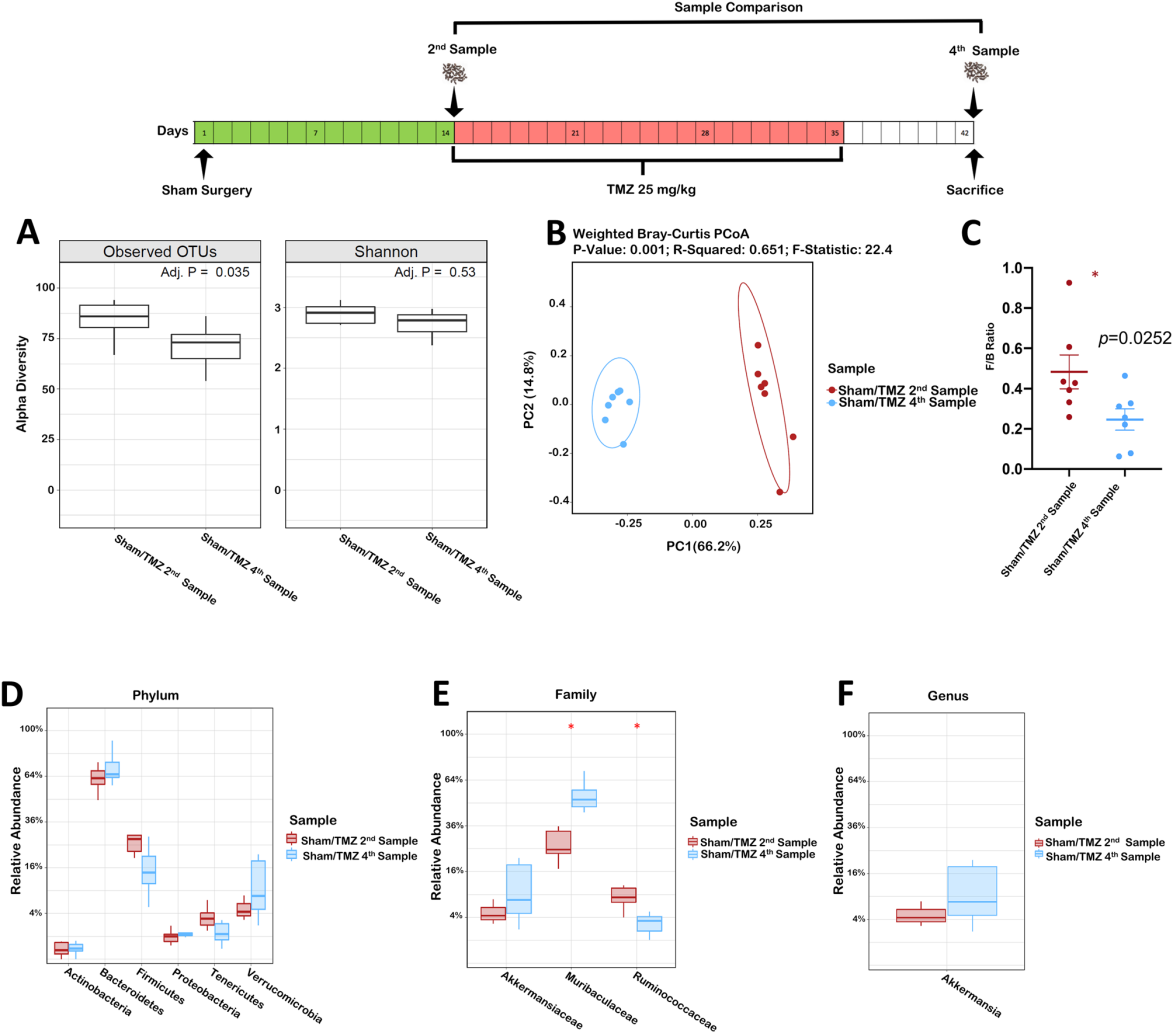
(**A–F**) Temozolomide alters the mouse gut microbiome. Comparison of Sham/TMZ 2nd and 4th samples (n = 7). (**A**) Observed OTU and the Shannon alpha diversity index. (**B**) Beta diversity Weighted Bray–Curtis PCoA. (**C**) *Firmicutes* to *Bacteroides* ratio. (**D**) Relative abundance taxa summary at the Phylum level. (**E**) Relative abundance taxa abundance at the family level. (**F**) Relative abundance of *Akkermansia* at the genus level.

After demonstrating a glioma induced GM dysbiosis and that this was not observed in the setting of TMZ, we aimed to determine the TMZ effect on mouse GM. We evaluated this in non-tumor bearing mice (Sham/TMZ group). Samples were compared prior (2nd Sample) and after TMZ therapy (4th Sample/sacrifice), Fig. 2. Observed OTUs were different between samples (*p* = 0.035), however, the Shannon index showed comparable microbiome community richness and diversity (*p* = 0.53, Fig. 2A). TMZ administration for three-weeks resulted in a clear separation of the microbiome beta diversity (*p* = 0.001, Fig. 2B). In addition, a decrease in the F/B (*p* = 0.02, Fig. 2C) was observed. However, there were no significant differences in *Verrucomicrobia*, *Akkermansiaceae*, or *Akkermansia* (Fig. 2D–F). Importantly, there was no weight loss following TMZ administration (pre-TMZ 27 ± 0.97 vs. post-TMZ 27.29 ± 0.94, *p* = 0.27). *Muribaculaceae* and *Ruminococcaceae* families increased and decreased following TMZ, respectively (*p* = 0.01 and 0.04, Fig. 2E). In addition, TMZ altered gut permeability in aged mice as measured by the dextran assay (*p* < 0.05, Supplementary Fig. S6). Altogether, these results show that TMZ itself causes dysbiosis in the GM, but without affecting *Verrucomicrobia* or *Akkermansia* abundance.

### Effect of glioma and temozolomide in patients with glioma

**Figure 3:**
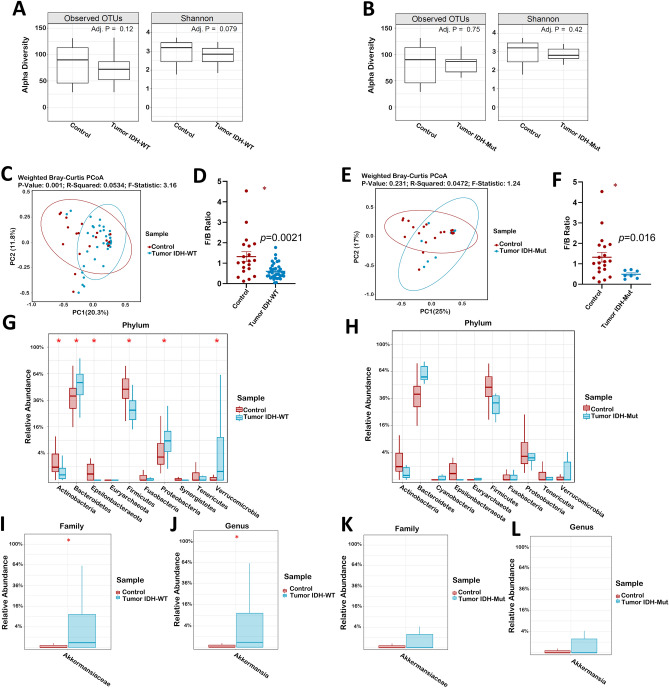
IDH-WT and IDH-Mut gliomas display different effects on the human gut microbiome. Comparison of patients' gut microbiome at diagnosis, based on *IDH* gene status (IDH*-*WT, n = 39 and IDH*-*Mut, n = 8) against controls (n = 21). (**A**,**B**) Observed OTU and the Shannon alpha diversity index between controls and IDH-WT or IDH-Mut gliomas patients. (**C**) Beta diversity Weighted Bray–Curtis PCoA of IDH*-*WT gliomas. (**D**) *Firmicutes* to *Bacteroides* ratio of IDH-WT gliomas. (**E**) Beta diversity Weighted Bray–Curtis PCoA of IDH*-*Mut gliomas. (**F**) *Firmicutes* to *Bacteroides* ratio of IDH-Mut gliomas. (**G**,**H**) Relative abundance taxa summary at the Phylum level of IDH*-*WT and IDH*-*Mut gliomas. (**I**) Relative abundance of IDH*-*WT glioma in *Akkermansiaceae* at the family level (**J**) Relative abundance of IDH*-*WT glioma in *Akkermansia* at the genus level. (**K**) Relative abundance of IDH*-*Mut glioma in *Akkermansiaceae* at the family level. (**L**) Relative abundance of IDH*-*WT glioma in *Akkermansia* at the genus level.

After our mice experiments demonstrating the effects of glioma development and TMZ in the GM of mice, we evaluated if this effects were also observed in glioma patients compared to healthy controls. Eighty-three stool samples from 53-glioma patients were prospectively collected. 47-samples at diagnosis (39 IDH-wildtype and 8 IDH-mutant), and 18 matching pairs before and after chemoradiation. Tumors were classified according to the WHO 2016 classification^23^. Median age was 55.5-years and 64% were males. Median pre-operative Karnofsky Performance Status (KPS) was 70, and most patients (n = 39, 74%) were treated with the Stupp protocol^24^, Table 1. Median follow-up was 7.0 months. No differences were observed in OTUs and Shannon index between controls and IDH-WT (Fig. 3A) or IDH-Mut (Fig. 3B) glioma at diagnosis. The PCoA plot displayed separate clustering between controls and glioma IDH-WT (*p* = 0.001, Fig. 3C), but not in IDH-Mut patients (*p* = 0.23, Fig. 3E). F/B ratio demonstrated significant differences between IDH*-*WT and IDH*-*Mut patients compared to controls (*p* = 0.0021 and *p* = 0.016, respectively, Fig. 3D–F). Interestingly, marked differences were observed at the phylum level in IDH-WT patients compared to controls, with increased *Bacteroidetes, Proteobacteria*, and *Verrucomicrobia* (*p* = 0.02, 0.02 and 0.01, respectively, Fig. 3G), however, these were not observed in IDH-Mut patients (Fig. 3H). *Akkermansiaceae* (*p* = 0.035, Fig. 3I), and *Akkermansia* (*p* = 0.037, Fig. 3J) were significantly higher in IDH-WT patients compared to controls. In IDH-Mut patients, there was no difference in *Akkermansiaceae* (*p* = 1.0, Fig. 3K), and *Akkermansia* (*p* = 1.0, Fig. 3L). Twenty-one control samples were analyzed and compared to samples from glioma patients at initial diagnosis. The control samples demographic characteristics are demonstrated in Supplementary Table S1.

**Table 1:** Clinical Characteristics and Demographics of Glioma Patients (N = 53).

Variable	N (%)
**Age group at diagnosis**	
18–54	26 (49)
> 55	27 (51)
**Sex**	
Male	34 (64)
Female	19 (36)
**Race/ethnicity**	
White/Caucasian	26 (49)
African American	6 (12)
Hispanic	15 (28)
Asian/Pacific Islander	5 (9)
Others	1 (2)
**Pre-operative KPS**	
70 or more	31 (58)
< 70	22 (42)
**BMI**	
< 18.5	3 (6)
18.5–24.9	12 (23)
25–29.9	21 (40)
30–34.9	9 (17)
35–39.9	4 (7)
> 40	4 (7)
Tumor location*	
Frontal	24 (45)
Parietal	16 (30)
Temporal	25 (47)
Occipital	8 (15)
Insular/Paralimbic	21 (40)
Brainstem, thalamus, and basal ganglia	8 (15)
Intraventricular	4 (7)
Butterfly	4 (7)
Multifocal	4 (7)
**Diagnosis**	
Glioblastoma IDH-wild type	40 (75)
Glioblastoma IDH-mutant	6 (12)
Anaplastic astrocytoma IDH-wild type	1 (2.5)
Anaplastic oligodendroglioma IDH-mutant 1p19q co-deleted	1 (2.5)
Diffuse astrocytoma IDH-wild type	2 (4)
Diffuse astrocytoma IDH-mutant	3 (6)
Tumor volume (cc) **	
16.4 -113.3	14 (26)
113.3—178.9	13 (24.67)
178.9 -219.3	13 (24.67)
219.3–374.4	13 (24.67)
**Radiological extent of resection (1st surgery)**	
GTR	19 (36)
NTR	12 (23)
STR	18 (34)
Biopsy	4 (7)
**Temozolomide**	
Yes	39 (74)
No	11 (20)
Not available	3 (6)
**Radiotherapy**	
Yes	39 (74)
No	11 (20)
Not available	3 (6)
Other treatments***	
Yes	10 (19)
No	42 (81)
**Progression**	
Yes	20 (38)
No	33 (62)
**Survival status**	
Death	5 (9)
Alive	48 (91)

*KPS* Karnofsky Performance Score, *BMI* body mass index, *IDH* isocitrate dehydrogenase, *GTR* gross-total resection, *NTR* near-total resection, *STR* Sub-total resection.

*More than one location was listed if the tumor involved more than one lobe.

**Volumetric analysis included the enhancing tumor, necrosis, and T2-Flair changes.

***Other treatments included: Depatuxizumab Mafodotin n = 5 (Clinical Trial), Bevacizumab n = 3, Novo TTF (Tumor Treating Fields) n = 3, Immunotherapy n = 2, CPT-11 n = 1, Laser Interstitial Thermal Therapy—LITT n = 1.

TMZ effect on the microbiome was determined by comparing samples before and after TMZ in 18-patients (14-IDH-WT and 4-IDH-Mut gliomas). Our results showed that *Verrucomicrobia*, *Akkermansiaceae*, and *Akkermansia* abundance trend to decrease following TMZ administration, however, no alpha diversity, F/B ratio, and PCoA changes were observed (Supplementary Fig. S7).

Preliminary progression-free survival (PFS) analysis in *Akkermansia* positive (12) vs *Akkermansia* negative (14) GBM patients demonstrated a non-significant difference between groups (8.8 months vs 6.3 months, *p* = 0.842), respectively.

## Discussion

In the present study, we investigated GM changes in mice following both tumor development and TMZ chemotherapy, as well as in a prospective cohort of newly diagnosed glioma patients. Our results demonstrate, significant glioma-induced change in the F/B in young mice following glioma development. Furthermore, glioma development was associated with a significant decrease in *Firmicutes* and an increase in the *Verrucomicrobia* phylum of which the predominant genus is *Akkermansia*^20^. The GM of aged mice differed at baseline from young mice, including changes in *Firmicutes, Bacteroidetes*, *Actinobacteria,* and *Tenericutes*. Although, *Firmicutes* and *Bacteroidetes* changes have been previously demonstrated to occur with age^22^, little is known about the changes of *Actinobacteria* and *Tenericutes* with aging. Despite these differences, similar results were observed in both young and aged mice following tumor development and TMZ treatment.

Our findings in both young and aged were consistent, including a clear clustering in the weighted Bray–Curtis distances of the GM, decreased F/B ratio, and the relative increased of the phylum *Verrucomicrobia* with a resulting increase in the genus *Akkermansia* and its more common species *Akkermansia muciniphila (AM).*

*AM* a mucin-degrading bacteria has been found to be inversely associated with inflammation, diabetes, obesity, and cardio-metabolic diseases and has been described as the next-generation beneficial probiotic^5,15,25^. *AM* converts mucin to short-chain fatty acids, including acetate and propionate, which may mediate its immunoregulatory effects, conferring both regulatory and inflammatory properties via interactions with the host immune system^26^.

Recent studies have demonstrated that *A. muciniphila* is increased in stroke, Parkinson’s disease, and untreated MS^15–19^. These reports suggest that *Akkermansia* abundance correlates with interferon signaling, T-cell and monocyte gene expression, namely NF-kB, that have been implicated in disease pathogenesis^17^. Furthermore, *Akkermansia* abundance has been positively correlated to pro-inflammatory pathways including upregulation of genes involved in antigen-presentation, B and T-cell receptor signaling, activation of complement, and coagulation cascades^17,26^. These pro-inflammatory features may be related to its ability to degrade mucus, leading to gut barrier breakdown and increased exposure of resident immune cells to microbial antigens^26^. Similar signaling pathway (IFN-ƴ and IL-2) alterations have been implicated in CNS toxicity, blood–brain barrier (BBB) disruption, and vasogenic brain edema in glioma^27^.

In our study, the *Akkermansia* population significantly increased following glioma development both in mice and in IDH-WT glioma patients. The similarity of findings across humans and mice do not come as a surprise as GL261 cells used in this study are IDH-WT^28^. Considering that IDH-WT and IDH-Mut gliomas are distinct molecular subgroups with IDH-WT being more aggressive and having a worse prognosis^29^, it was not surprising to observe dysbiosis particularly in the IDH-WT human cohort.

Recent studies demonstrate how the GM composition in cancer patients influences the response to ICI. Importantly, *A. muciniphila* abundance correlated with clinical response to PD-1/PD-L1 therapy^12^. Despite ICI success in many solid cancers, little efficacy has been seen in GBM^30,31^. Studies evaluating if certain GM taxa, including *Akkermansia,* may enhance specific therapies previously failed in GBM are warranted. Moreover, the potential of therapeutic modulation of the microbiome by probiotics/ fecal transplant to expand therapeutic options for glioma deserves further study.

TMZ, an oral agent with a unique pharmacological profile, is considered the standard of care for adjuvant therapy, in combination with radiotherapy^24,32^. Prior studies have evaluated the effects of chemotherapeutic agents on the GM^33^. Cyclophosphamide has been found to cause disruption of the intestinal barrier, translocation of commensal bacteria, and small intestine microbiome alterations^34^. Despite its well-established safety and ease of administration, the TMZ effects on the GM remain unknown.

Our mice results demonstrate that TMZ alters the GM with clear separation in weighted Bray–Curtis distances and a significant decrease in the F/B following oral TMZ. In addition, levels of , *Muribaculaceae* and *Ruminococcaceae* increased and decreased respectively. The later, has been associated with maintenance of gut health^35^. A higher relative abundance of *Ruminococcaceae* has been found in GM of melanoma responders versus non-responders to PD-1 immunotherapy. Furthermore, higher effector CD4^+^ and CD8^+^ T-cells frequency and a preserved cytokine response coincided with higher *Ruminococcaceae* levels in anti-PD-1 responders, while those who were non-responders displayed higher *Bacteroidales* levels^13^.

*Muribaculaceae* abundance has been correlated with increased short-chain fatty acids (SCFAs) levels^36^, which affect microglial density, morphology, and maturity^5,36^. Additional studies of SCFA producing bacteria, fecal metabolites, neurotransmitters and their interactions with the immune system may allow for potential microbiome manipulations in glioma patients.

*Verrucomicrobia* and *Akkermansia* increased abundance following tumor implantation in mice were not observed following TMZ treatment. Interestingly, TMZ by itself (Sham/TMZ) did not alter *Verrucomicrobia* or *Akkermansia* levels in mice. We hypothesize that the observed *Verrucomicrobia*/*Akkermansia* reduction in the tumor/TMZ group is the result of tumor control. Similar to our findings in mice, *Akkermansia* increased abundance in glioma patients demonstrated a decreasing trend following TMZ treatment (Supplementary Fig. S7). Understanding the interactions between glioma and the GM is vital as a particular microbiome composition may enhance the effects of existing therapies (chemotherapies, targeted therapies, immunotherapies), which have not yet been effective in glioma^30,31,37^.

Despite our robust findings, there are several limitations to our study. First, we did not find a relationship between the GM, clinical factors, and survival in mice or humans, which could be secondary to the limited number of patients and short follow-up in this preliminary study. Future studies with larger cohorts and longer follow-up are needed to investigate possible clinical associations. Second, TMZ effects in humans may be influenced by cohort-specific cofounders, small sample size, and radiotherapy treatment^32,38^. Third, glioma patients received dexamethasone prior to surgery, which could potentially affect the GM^39^. Lastly, while our microbiome analysis using 16S rRNA is useful for providing taxonomic information, shotgun metagenomics, metatranscriptomic and metabolomic profiling of FS may reveal changes that would complement phylogenic and taxonomic data. Additionally, further experiments are needed to evaluate the effect of other intracranial tumors on the GM to assess if the findings in our study are specific to glioma development. In addition, the short follow-up of this preliminary study might preclude us to identify specific microbial taxa that correlated with survival. Even though our experiments fell short of establishing a relationship between the microbiome and clinical parameters, there have been numerous studies that have shown a correlation between *Akkermansia* and pro-inflammatory genes in macrophages and T-cells with downstream effects in IFN-ƴ and IL-2; which in turn cause BBB disruption and cerebral edema in glioma patients. This may be a plausible explanation for the increased level of *Akkermansia* in our mouse-model and in IDH-WT glioma patients.

## Conclusions

This study demonstrates for the first time the relationship between the gut-brain axis, glioma development, and TMZ in both mice and humans. We demonstrated that *Verrucomicrobia* phylum and *Akkermansia* genus abundance increase after glioma growth in a mouse-tumor model, which was not observed in the setting of TMZ treatment. These findings appear to be translational, as humans with IDH-WT gliomas also show similar microbiome changes when compared to controls. Further studies to evaluate the relationship between the gut-brain axis and glioma are needed to address the role that the GM, especially the genus *Akkermansia* plays in this disease.

## Methods

### Animals

**Figure 4:**
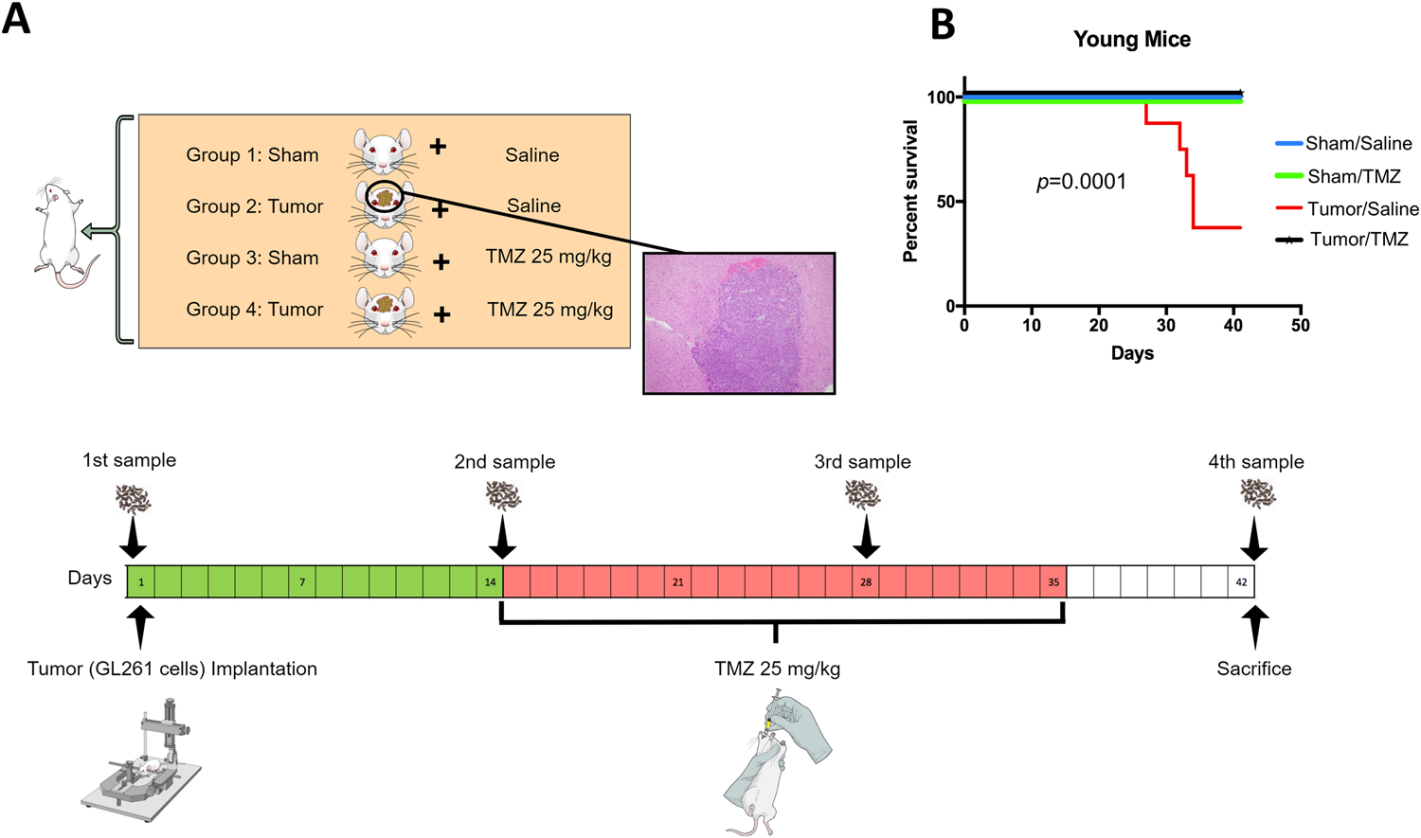
Experimental design and stool sample collection. (**A**) Twenty-eight male C57BL/6 mice were randomly divided into four groups: Sham/ Saline (N = 7), Tumor/ Saline (N = 6), Sham/ TMZ (N = 7), and Tumor/ TMZ (N = 8). The tumor was implanted using GL261 mouse cells. Two weeks post tumor implantation, mice were treated orally by either saline or TMZ 25 mg/kg for three weeks, five days per week. Stool samples were collected periodically as depicted in the timeline. (**B**) Kaplan–Meier survival curve using the Log-rank test of the 4 groups, in which mortality was significantly higher in the tumor/saline group (*p* < 0.001) compared to the other three groups.

Six- to eight-week-old male C57BL/6 mice from the Jackson Laboratory (Bar Harbor, ME, USA) and 20-month-old male C57BL/6 mice from the National Institute on Aging were used. Thirty young mice were assigned to four groups: G1 Sham/Saline (n = 7), G2 Tumor/Saline (n = 8), G3 Sham/TMZ (n = 7), and G4 Tumor/TMZ (n = 8). Tumor implantation was performed on day 0. On day 14^40–42^, mice were orally gavaged with TMZ (25 mg/kg) or saline daily, 5 days per week for 3 weeks. FS were collected and stored in sterile tubes at -80 °C prior to tumor implantation (1st sample), before and after the initiation of TMZ/Saline treatment (2nd and 3rd sample), and at sacrifice, on day 42 or at mice death if this was prior to sacrifice (4th sample) (Fig. 4)^43^. Brains implanted with GL261 cells were examined to confirm tumor growth^44^. Gut permeability assay was performed as previously described^45^. All experimental work was approved by the Laboratory Animal Medicine and Care at the University of Texas Health Science Center at Houston institutional review board (IRB) in compliance with the Guide for the Care and Use of Laboratory Animals.

Twelve aged mice were assigned to two groups: G1Tumor/Saline (n = 6) and G2 Tumor/TMZ (n = 6). However, both group's numbers were reduced due to mortality after tumor implantation (G1 n = 3, G2 n = 4). Tumor implantation, drug dosing, and stool collection followed the same protocol as young mice.

### Microbial DNA extraction and the 16S rRNA gene sequencing

16S rRNA gene sequencing methods were adapted from the methods developed for the Earth Microbiome Project and NIH-Human Microbiome Project^46–49^. Subsequent analysis steps were performed at the Alkek Center for Metagenomics and Microbiome Research (CMMR) at Baylor College of Medicine. 16Sv4rRNA sequences were clustered into Operational Taxonomic Units (OTUs) at a similarity cutoff value of 97% using the UPARSE algorithm^50^ and mapped to an optimized version of the SILVA Database^51^ containing only sequences from the v4 region of the 16S rRNA gene to determine taxonomies. A custom script constructs an OTU table from the output files for downstream analyses using a visualization toolkit also developed at the CMMR named ATIMA (Agile Toolkit for Incisive Microbial Analyses).

### Patients’ characteristics and fecal sample collection

The prospective study was conducted from January 2018-July 2019. Patients with newly diagnosed glioma who provided a pre-surgical FS were included. Patients with recent antibiotic exposure (30-days), < 18-years, other cancers, and gastrointestinal diseases were excluded. Patients’ demographics, clinical, histological, and follow-up information, were obtained from the electronic medical record and stored in a REDCap database, which was later exported for analysis (Table 1). Patients underwent biopsy or maximum safe tumor resection at the discretion of the treating neurosurgeon followed by chemoradiotherapy^24^. *IDH1* p.R13H was assessed by immunohistochemistry. FS were collected from patients at three-time points: before resection (Pre-Sx) / prior to cephalosporin per-protocol; before and after 6-weeks of chemoradiation (Pre-Tx and Post-Tx*).* To minimize confounding factors known to impinge on the human microbiome composition^32,38^, twenty-one FS were utilized as controls. FS for DNA extraction and microbiome sequencing were collected using microbial collection and stabilization kits (OMNIgene Gut; DNA Genotek). This prospective human study was approved by our institution IRB and it was in accordance with the 1964 Helsinki Declaration and its later amendments.

### Statistical analysis

The ATIMA software was used to perform the downstream analyses. The significance of categorical variables was determined using the Mann–Whitney/Kruskal–Wallis test. All *p*-values were two-sides and adjusted for multiple comparisons with the FDR-algorithm.

The F/B was calculated by Mann–Whitney/paired t-test. This was evaluated as F/B represents the two most common phyla in the GM. Additionally, F/B ratio has been known to affect outcome in neurological diseases including stroke^22^. PFS of patients with 6-months or longer follow-up was plotted using the Kaplan–Meier method in GraphPad Prism (version 8.2.1 for Mac, GraphPad, CA, USA). Analysis in the aged-mice was performed using paired t-test, prior confirmation of normal distribution. Mice were randomly assigned to treatment groups. All analyses were performed by a blinded investigator. For a complete methodology description, see Supplementary Methodology.

### Ethical approval

All procedures performed in this prospective human study were approved by the institutional review board of The University of Texas Health Science Center at Houston and Memorial Hermann Hospital, Houston, TX and it was in accordance with the 1964 Helsinki Declaration and its later amendments.

### Consent to participate

Informed consent was obtained from all individual participants included in the study.

## Supplementary information

Supplementary Information.

## Data Availability

The datasets generated during and/or analyzed during the current study are available from the corresponding author on reasonable request.
