# Supplementary material for: Glioma and temozolomide induced alterations in gut microbiome
Source: Sci Rep. 2020 Dec 3;10:21002. doi: 10.1038/s41598-020-77919-w (PMC7713059; doi:10.1038/s41598-020-77919-w)
Supplement: Supplementary file 1 — Supplementary Information. [file 41598_2020_77919_MOESM1_ESM.pdf]

Glioma and Temozolomide Induced Alterations in Gut Microbiome

Anthony Patrizz, Ph.D.,[1, †] Antonio Dono, M.D.,[1,2 †] Soheil Zorofchian, Ph.D.,[1,2, †] Gabriella Hines, B.S.,[2] Takeshi Takayasu, M.D., Ph.D.,[1,2] Nuruddin Husein, B.S.,[1] Yoshihiro Otani, M.D., Ph.D.,[1] Octavio Arevalo, M.D.,[3] H. Alex Choi, M.D.,[1] Jude Savarraj, Ph.D.,[1] Nitin Tandon, M.D.,[1] Bhanu P. Ganesh, Ph.D.,[4] Balveen Kaur, Ph.D.,[1] Louise D. McCullough, M.D., Ph.D.,[4] Leomar Y Ballester, M.D., Ph.D.,[1,2,6*] and Yoshua Esquenazi, M.D.,[1,5,6*]

[1]Vivian L. Smith Department of Neurosurgery, [2]Department of Pathology and Laboratory Medicine, [3]Department of Diagnostic and Interventional Imaging, [4]Department of Neurology, [5]Center for Precision Health, The University of Texas Health Science Center at Houston, McGovern Medical School, Houston, Texas, USA, [6]Memorial Hermann Hospital-TMC., Houston, Texas, USA. [†]Contributed equally as first authors.

Supplementary Methodology

Animals

Six- to eight-week-old male C57BL/6 mice were obtained from the Jackson Laboratory (Bar Harbor, ME, USA) and 20-month-old male C57BL/6 mice from the National Institute on Aging and were allowed to acclimate for a minimum of four weeks before use. All mice were housed in a temperature- and humidity-controlled vivarium, 5 per cage (11” L, 6” W, 6” H) with a 12-hour light/dark schedule with *ad libitum* access to food and water. All experimental work was approved by the Laboratory Animal Medicine and Care at the University of Texas Health Science Center at Houston institutional review board (IRB) in compliance with the Guide for the Care and Use of Laboratory Animals.

Thirty young mice were assigned to four groups: Group 1 Sham with saline (vehicle) (n=7), Group 2 Tumor with saline (n=8), Group 3 Sham with TMZ (n=7), and Group 4 Tumor with TMZ (n=8). All animals were housed in cages based on their treatment group. Tumor implantation was performed on day 0. Beginning on day 14, mice were orally gavaged with TMZ (25mg/kg) or saline daily, 5 days per week (2 days off) for 3 weeks. Fecal samples were collected and stored in sterile tubes at -80℃ prior to tumor implantation (1st sample), before the initiation of TMZ/Saline treatment (2nd sample), after (3rd sample) treatment, and 4th at sacrifice or death (if this was prior to sacrifice). Mice were individually placed in clean cages without bedding for stool collection. Any animal displaying signs of significant weight loss (>10% from baseline) or immobility were euthanized and tissue collected. All surviving animals were sacrificed on day 42 with 0.1mL/10g body weight dose of tribomoethanol (Sigma-Aldric, MO, USA) dissolved in 2-Methyl-2-Butanol. Stool samples were collected from the large intestine (4th sample) at the time of sacrifice (**Figure 1**) and kept frozen at -80° C. Fresh major organs (including the brain) were stored in formalin and processed for paraffin sections and H & E staining. Brains that were implanted with GL261 cells were macroscopically and histologically examined by a certified neuropathologist (LYB) to confirm the presence of tumor for further analysis. Two mice were excluded from the tumor with saline group due to a lack of tumor development. Therefore, all the results of group 2 (tumor/saline) were analyzed with 6 mice.

Twelve aged mice were assigned to two groups: Group 1 tumor with saline (n=6) and group 2 tumor with TMZ (n=6). However, both group's numbers were reduced due to mortality within a few days after tumor implantation, thus they were excluded from further study (Group 1 n=3, Group 2 n=4). Tumor implantation, drug dosing, and stool collection followed the same schedule as young mice.

GL261 mouse model of glioma

For intracranial mouse xenograft studies, both young and aged mice were implanted with GL261 cell lines maintained in our laboratory. Briefly, GL261 cells were cultured in Dulbecco’s Modified Eagle's Minimal with 10% FBS, penicillin, and streptomycin in a humidified atmosphere with 5% CO2 at 37°C. Mice were anesthetized and stabilized in a stereotactic frame, a burr hole was drilled 2mm lateral and 1mm anterior to bregma in the right hemisphere, to a depth of

3.5mm.[20] GL261 (1×10^5 cells) in 2μl of Hank's buffered salt solution (HBSS) or the same amount of HBSS were implanted over 5 minutes using autoinjectors as a tumor-bearing group or sham group respectively.

Temozolomide

100mg of Temozolomide (Sigma-Aldrich, MO, USA) was dissolved in 1.5ml of DMSO (Sigma-Aldrich, MO, USA) and sonicated three times. The solution was further dissolved in 38.5ml of sterile saline for a final working concentration of TMZ 2.5mg/ml.

Gut Permeability Assay

On day 42, aged mice were fasted for 5 hours prior to sacrifice. One hour prior to sacrifice mice were orally gavaged with 6mg/10g body weight of 4kDa FITC dextran (Sigma-Aldrich, MO, USA), DMSO was used as control to ensure that the differences were not due to solvent of the drug. One hour later blood samples were collected, centrifuged at 4 C at 3000g for 6 minutes and stored at -80 C until use. Samples were analyzed using a fluorescence spectrometer at an excitation wavelength of 428nm and an emission wavelength of 535nm.[21]

Microbial DNA extraction and the 16S rRNA gene sequencing

16S rRNA gene compositional analysis provides a summary of the composition and structure of the bacterial component of the microbiome. Genomic bacterial DNA extraction methods were optimized to maximize the yield of bacterial DNA while keeping background amplification to a minimum. 16S rRNA gene sequencing methods were adapted from the methods developed for the Earth Microbiome Project and NIH-Human Microbiome Project.[22–24] Briefly, bacterial genomic DNA was extracted using the Qiagen MagAttract Power Soil DNA Kit. The 16S rRNA V4 region was amplified by PCR and sequenced on the MiSeq platform (Illumina, Inc. CA, USA) using the 2x250 bp paired-end protocol yielding pair-end reads that overlap almost completely. The primers used for amplification contain adapters for MiSeq sequencing and single-index barcodes so that the PCR products may be pooled and sequenced directly,[24] targeting at least 10,000 reads per sample. CMMR16S (variable region 4 [v4]) rRNA gene pipeline data incorporates phylogenetic and alignment-based approaches to maximize data resolution. The read pairs are demultiplexed based on unique molecular barcodes added via PCR during library generation, then merged using USEARCH v7.0.1090.[25] The subsequent analysis steps of the pipeline leverage custom analytic packages developed at the Alkek Center for Metagenomics and Microbiome Research (CMMR) at Baylor College of Medicine to produce summary statistics and quality control measurements for each sequencing run, as well as multi-run reports and data-merging capabilities for validating built-in controls and characterizing microbial communities across large numbers of samples or sample groups. 16Sv4rRNA sequences are clustered into OTUs at a similarity cutoff value of 97% using the UPARSE algorithm.[26] OTUs are subsequently mapped to an optimized version of the SILVA Database[27] containing only sequences from the v4 region of

the 16S rRNA gene to determine taxonomies. Abundances are recovered by mapping the demultiplexed reads to the UPARSE OTUs. A custom script constructs an OTU table from the output files generated in the previous two steps for downstream analyses using a visualization toolkit also developed at the CMMR named ATIMA (Agile Toolkit for Incisive Microbial Analyses).

Patient characteristics and fecal sample collection

This prospective study was approved by the institutional review board of our institution. The study was conducted from January 2018 to July 2019. Patients in whom a fecal sample was obtained prior to surgical resection of a newly diagnosed glioma at our institution were included in the study. Patients with recent antibiotic exposure (30 days) for other related conditions, under 18-year of age, history of cancer, and presence of gastrointestinal diseases (e.g. inflammatory bowel disease) were excluded from the study. All tumors were examined by H&E and immunohistochemistry by a board-certified neuropathologist. Patients' demographics, clinical and follow-up information, including age, gender, race, Karnofsky performance score (KPS), body mass index (BMI), diagnosis according to the 2016 WHO classification of brain tumors, tumor volume, radiographic and volumetric extent of resection (EOR), adjuvant therapy, progression, and survival were obtained from the electronic medical record (EMR) and stored in a prospective REDCap database, which was later exported for data analysis (Table 1). Patients underwent biopsy or maximum safe tumor resection at the discretion of the treating neurosurgeon (YE, NT) followed by radiation therapy with concomitant TMZ (75mg/m2) in the majority of the cases. Following this, the maintenance dose of TMZ according to the Stupp protocol was implemented.[28] *IDH1* p.R13H immunohistochemistry was performed with the mutant protein-specific antibody (1:40; H09 monoclonal, Dianova, Hamburg, Germany) in a Dako Omnis (Agilent, California, USA) autostainer. The imaging analysis consisted of the volumetric measurement of three tumor components: Enhancing tumor, necrosis, and non-enhancing FLAIR hyperintensity. The enhancing tumor volume and necrosis volume were obtained from standard-of-care MRIs on routine post-contrast 2D or 3D T1-weighted images before and after the resection. FLAIR signal abnormality volume was measured on standard 2D or 3D T2-FLAIR images. Volumes were calculated using a semi-automated software (TeraRecon, Aquarius Intuition viewer). By using the threshold tool, the software selects volumes with a specific range of signal intensity values; then, the tumor boundaries are delineated utilizing the Region-of-interest (ROI) tool. The software automatically calculated the volume using computational algorithms. In the case of multiple lesions, the sum of all individual lesions was used. Fecal samples were collected from patients at three time points: before resection (Pre-Sx) and prior to prophylactic surgical single dose of cephalosporin per-protocol; prior to initiation of chemoradiation (Pre-Tx), which was collected at least 2-4 weeks after antibiotic administration to avoid antibiotic-induced biome changes;[33] and after completion of 6-weeks of chemoradiation (Post-Tx). Several common factors are known to impinge on the human microbiome composition including various environmental and social factors such as, lifestyle, diet, medications, place of residence, etc.[29,30] To minimize such

confounding factors and to increase our sample size, twenty-one fecal samples (9- household family members and 12- non-related individuals) were utilized as controls following the same exclusion criteria as enrolled patients (antibiotic exposure, under 18-year of age, history of cancer, and presence of gastrointestinal diseases).

Progression was analyzed in patients with more than 6 months of follow-up and with information regarding tumor progression or death. Fecal samples for DNA extraction and microbiome sequencing for most patients and control samples were collected using microbial collection and stabilization kits (OMNIgene Gut; DNA Genotek), however 12 controls samples were collected after rectal examination for a non-related condition with immediate freezing of the fecal sample at -80 C. After which, microbial DNA extraction and the 16S rRNA gene sequencing were performed.

Statistical analysis

The ATIMA (Agile Toolkit for Incisive Microbial Analyses) software was used to perform the downstream analyses. ATIMA is an R software suite combining publicly available packages (i.e. APE and VEGAN) and purpose written code to import sample data and identify trends in taxa abundance, alpha-diversity, and beta-diversity using weighted Bray-Curtis PCoA with sample metadata. The significance of categorical variables was determined using the non-parametric Mann-Whitney U test for two category comparisons or the Kruskal-Wallis test when comparing three or more categories. Correlation between two continuous variables is determined with R's base "lm" function for linear regression models, where p-values indicate the probability that the slope of the regression line is zero. PCoA plots employ the Monte Carlo permutation test to estimate p-values. All *p*-values were adjusted for multiple comparisons with the FDR algorithm.

Firmicutes to *Bacteroides* ratio (F/B) was calculated in normal Gaussian distribution (verified by the Anderson-Darling test, D'Agostino-Pearson omnibus normality test, Shapiro-Wilk normality test, and Kolmogorov-Smirnov normality test with Dallal-Wilkinson-Lillie for *p*-value) with paired t-test. Meanwhile, when no normal Gaussian distribution was present, the Mann-Whitney test was performed. PFS was calculated using Kaplan-Meier curves with Log-rank (Mantel-cox) test and the Gehan-Breslow-Wilcoxon test. Kaplan-Meier curves for PFS were generated using GraphPad Prism (version 8.2.1 for Mac, GraphPad, CA, USA). Analysis at phylum, family, and genus level in the aged-mice was performed using a paired t-test, due to the small sample size of this group prior to confirmation of normal distribution (Shapiro-Wilk normality test). All analysis was performed by a blinded investigator and mice were randomly assigned to treatment groups. OS analysis was not performed due to the short follow-up and status (alive) of most patients at the time of analysis (**Table 1).**

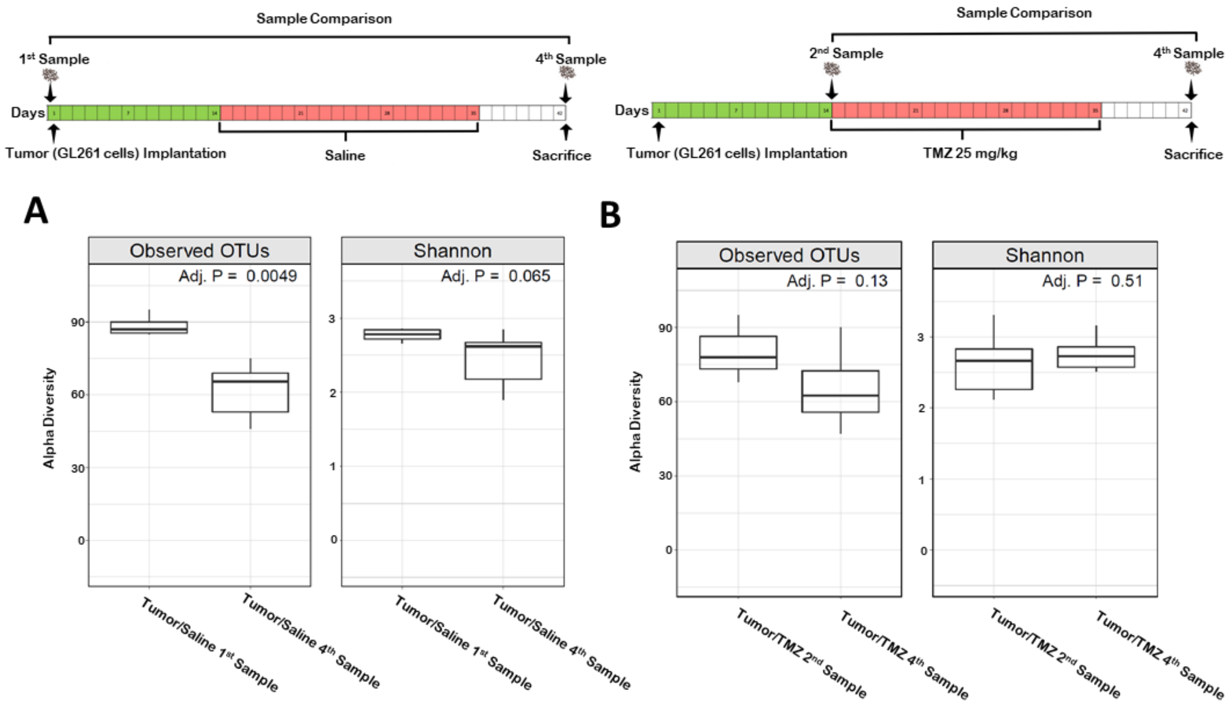

Supplementary Figure S1. Glioma induces dysbiosis in the mouse gut microbiome. A. Comparison of Tumor/Saline 1st and 4th samples (n = 6). Observe OTU and the Shannon alpha diversity index. B. Comparison of Tumor/TMZ 2nd and 4th samples (n = 8). Observe OTU and the Shannon alpha diversity index.

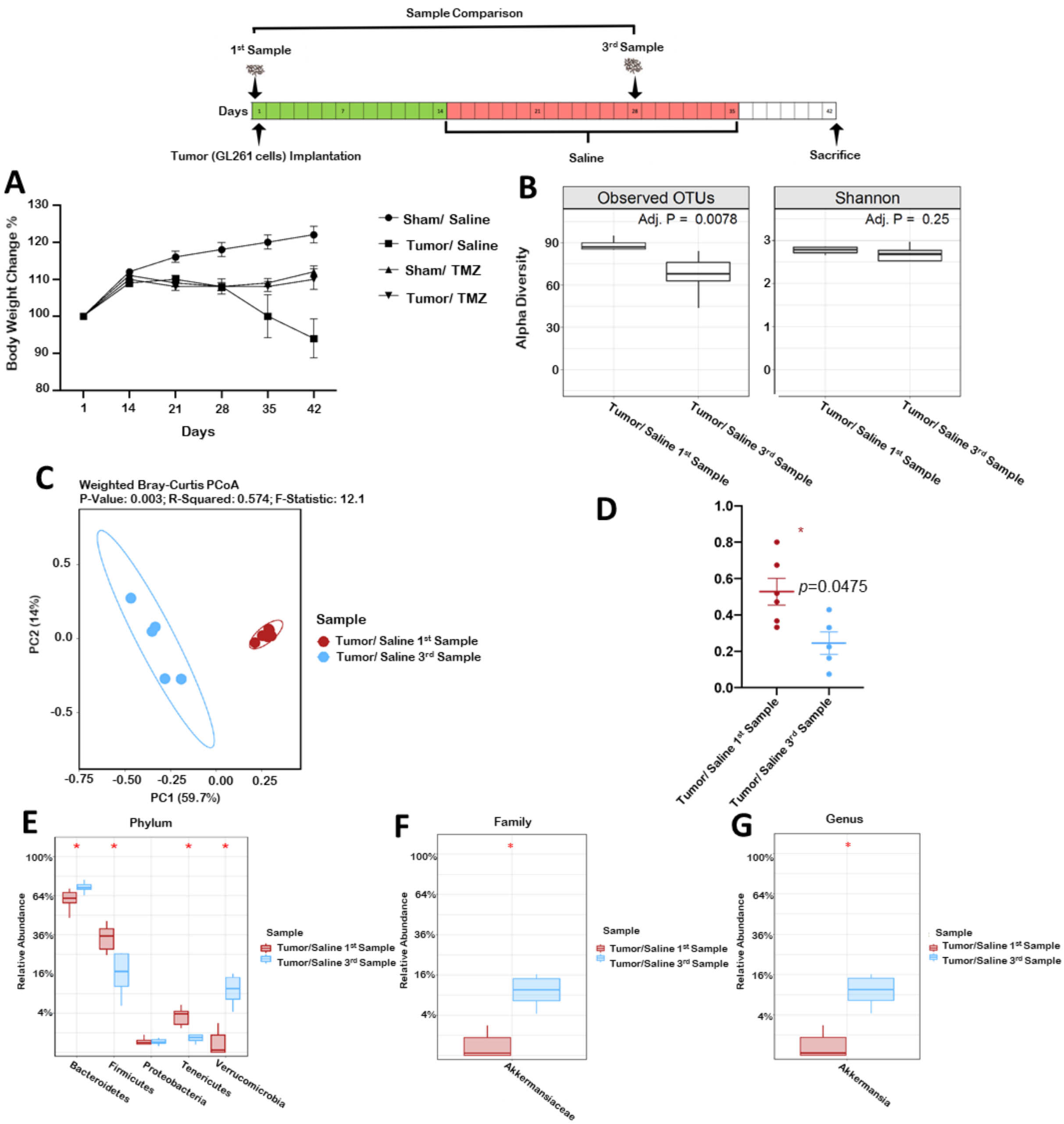

Supplementary Figure S2. Glioma induces dysbiosis in the mouse microbiome. Comparison of Tumor/Saline 1st and 3rd samples (n = 6) **A.** Comparison of weight loss of the different groups, in which no statistically significant difference can be seen at the 28 days between the different groups, when 3rd sample was collected (p= 0.56, F (3, 12) = 0.7052 in an ANOVA one-way analysis in GraphPad Prism. **B.** Observed OTUs were statistically significant *p*= 0.0078 with a non-significant Shannon diversity *p*=0.25. **C.** Beta Diversity Weighted Bray-Curtis PCoA *p*=0.003, R-Squared 0.574. F-statistic 12.1. **D.** F/B ratio (two-tailed paired t-test *p*= 0.0475 t=2 df=5). **E.** Relative abundance at the Phylum level, Verrucomicrobia, and Bacteroides were increased (*p*= 0.0289 and 0.0379) and Firmicutes and Tenericutes were decreased (*p*= 0.0289 and 0.0289, respectively). **F.** The relative abundance of *Akkermansiaceae* at the Family level (*p*=0.0189). **G.** Relative abundance of *Akkermansia* at the genus level (*p*= 0.0319).

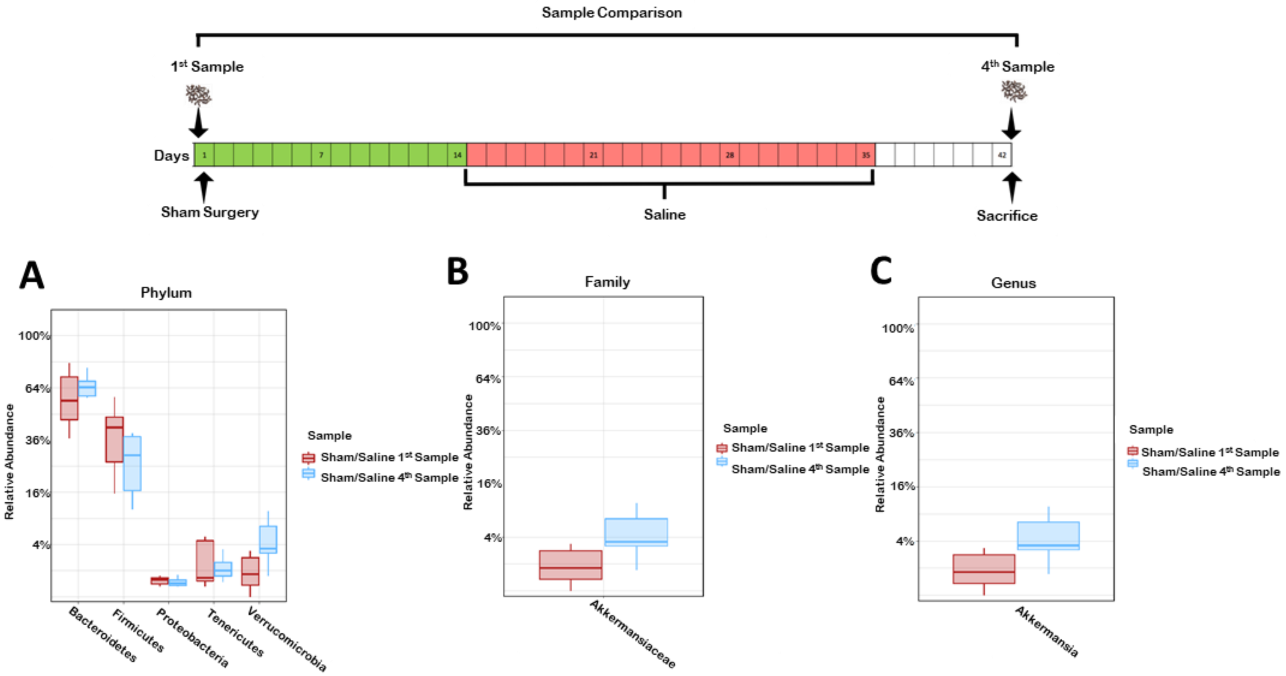

Supplementary Figure S3. A-C. Serial oral gavage does not alter the gut microbiome. No differences were observed between Sham/Saline 1st and 4th stool samples at the **(A)** Phylum level, especially in Verrucomicrobia phylum (p= 0.131), nor in *Akkermansiaceae* family p= 0.144 **(B)** or *Akkermansia* genus p= 0.131 **(C).**

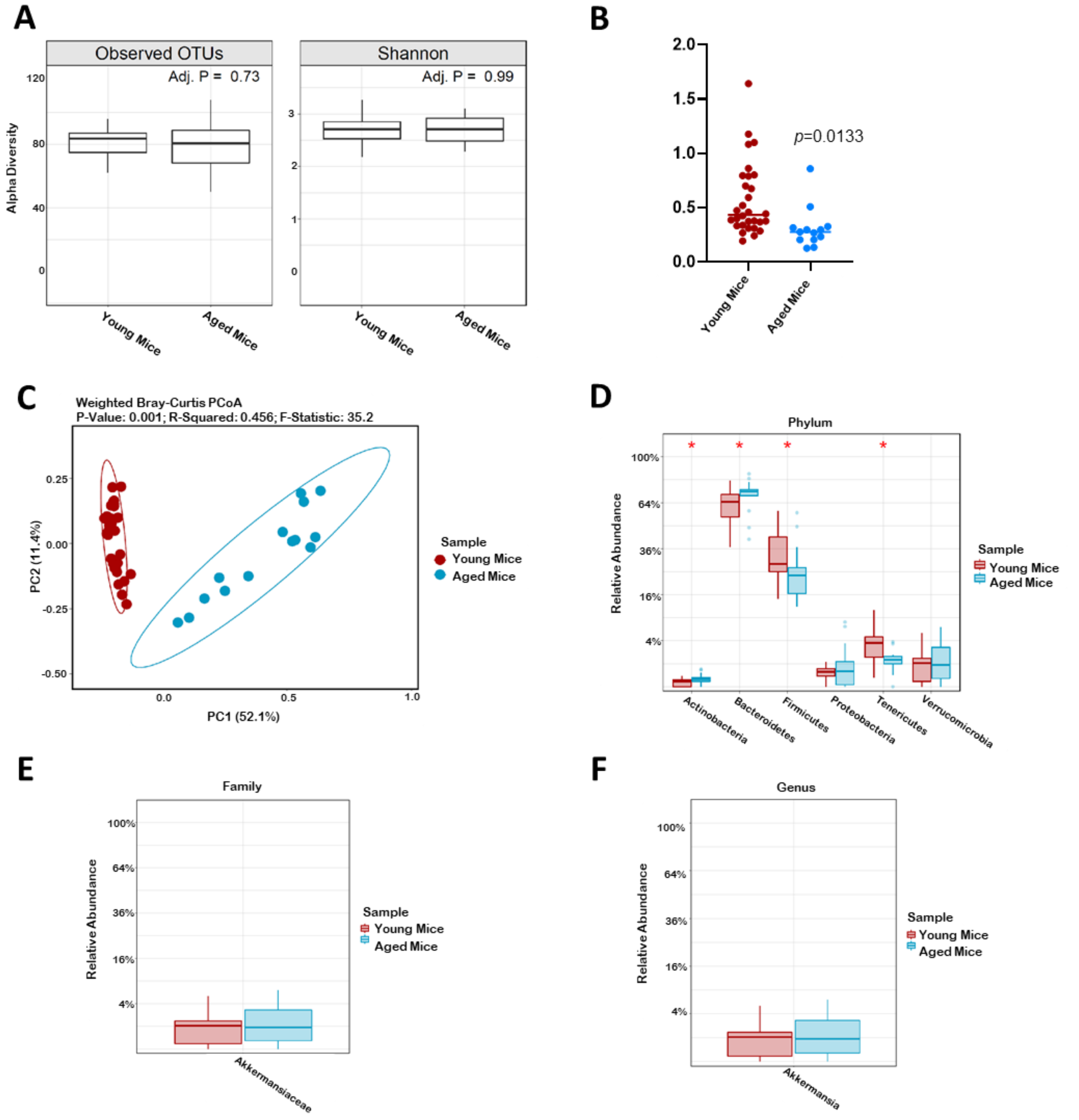

Supplementary Figure S4. Comparison of the gut microbiome of young and aged mice at baseline. (N=28 and 14, respectively) **A.** Alpha diversity demonstrates no significance difference in the observed OTUs p=0.73 or Shannon p=0.99. **B.** F/B ratio (Two-tailed unpaired T-test p=0.0133, t = 2.589, df = 41). **C.** Beta Diversity Weighted Bray-Curtis PCoA (p= 0.001, R-Squared 0.456. F-statistic 35.2) **D.** Relative abundance of Actinobacteria and Bacteroides at the Phylum level were increased (p= 0.0143 and 0.0296) and Firmicutes and Tenericutes were decreased (p= 0.0481 and 0.0102, respectively), but no difference in the levels of Verrucomicrobia were seen. **E.** At the Family level, *Akkermansiaceae's* relative abundance was not increased (p= 1.00). **F.** At the Genus level *Akkermansia's* relative abundance was not increased (P= 1.00).

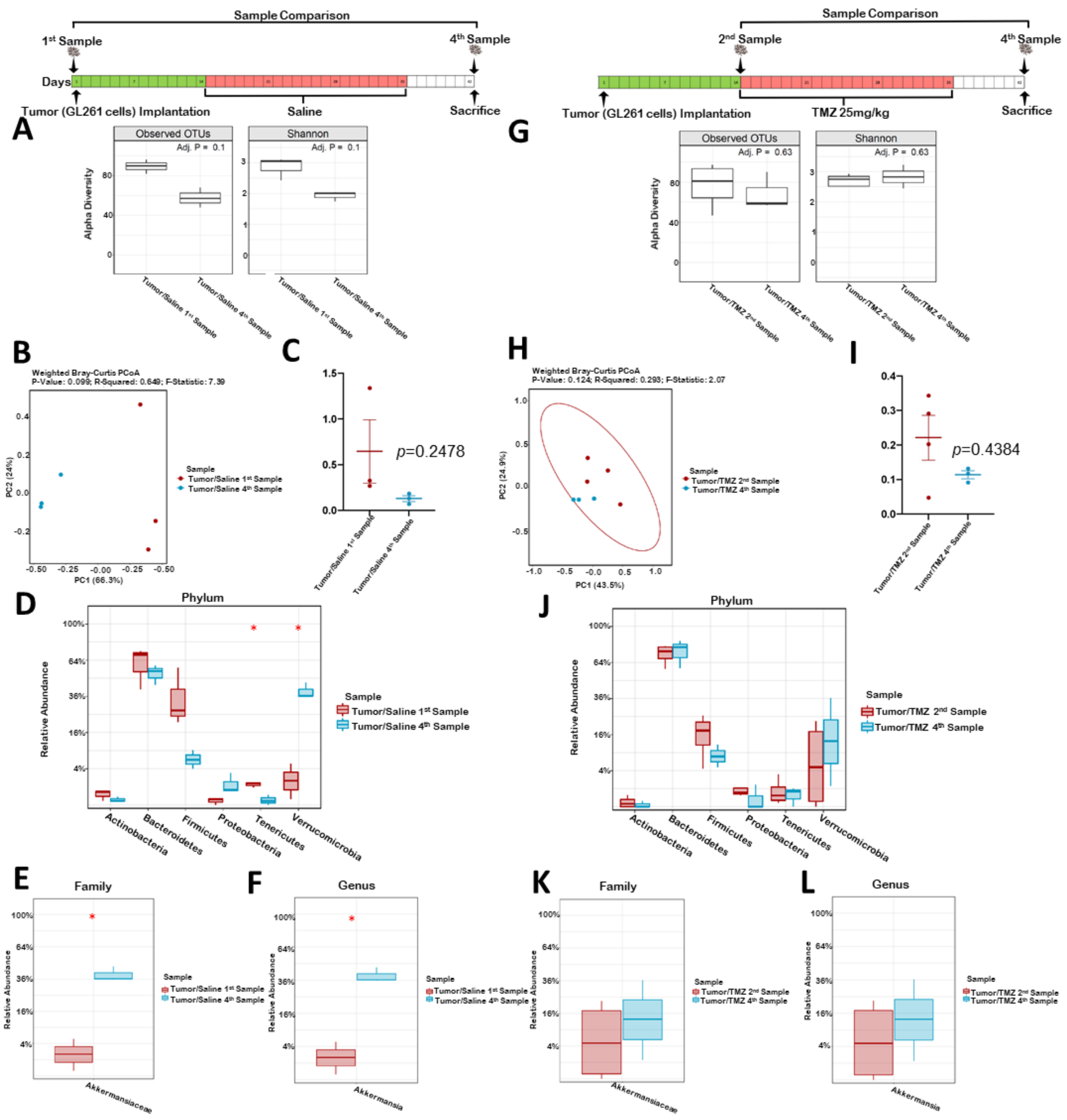

Supplementary Figure S5. A-F. Glioma induces dysbiosis in the aged gut microbiome. Comparison of Tumor/Saline 1st and 4th samples (n = 3) **A**. No significant difference between the observed OTU in alpha diversity (*p*= 0.1) or in the Shannon diversity (*p*= 0.1). **B.** Beta diversity Weighted Bray-Curtis PCoA (*p*= 0.099, R-Squared 0.649. F-statistic 7.39). **C.** F/B ratio (Two-tailed paired T-test p=0.2478, t = 1.615, df = 2). **D.** Phylum level relative abundance of Verrucomicrobia was increased (two-tailed paired t test, p=0.0123, t = 8.946, df = 2) and Tenericute was decrease (two-tailed paired t test, p=0.0355, t = 5.166, df = 2). **E.** The family level relative abundance of *Akkermansiaceae's* was increased (two-tailed paired t test, p= 0.0123, t = 8.946, df = 2). **F.** Genus level relative abundance of *Akkermansia's* was increased (two-tailed paired t test, p=0.0123, t=8.946, df=2). **G-L. Temozolomide depleted the Glioma-induced Dysbiosis in the aged gut microbiome.** Comparison of Tumor/TMZ 2nd and 4th samples (n = 4). **G.** No difference between the observed OTU in alpha diversity (*p*= 0.63) or in the Shannon diversity (*p*= 0.63). **H.** Beta-diversity Weighted Bray-Curtis PCoA (*p*=0.124, R-Squared 0.2931, F-statistic 2.07). **I.** F/B ratio (two tailed unpaired T-test p=0.4384 t = 0.96, df =2). **J.** At the Phylum level the relative abundance of Verrucomicrobia (*p*= 0.83). **K.** *Akkermansiaceae's* relative abundance (*p*= 1.00). **L.** Genus level relative abundance of *Akkermansia's* is not different between groups (*p*= 0.94).

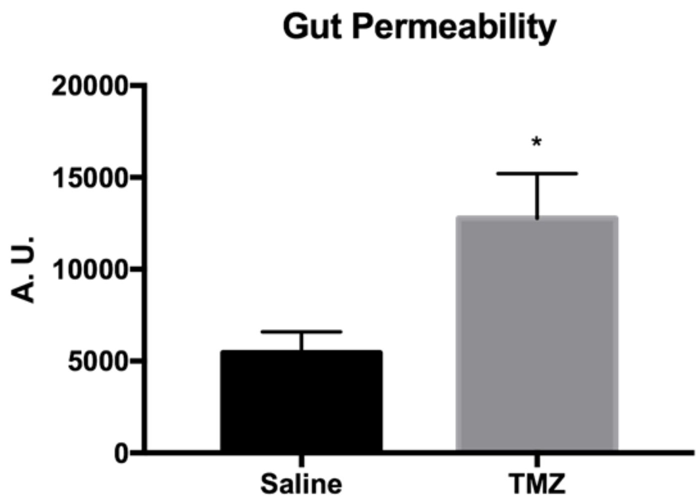

Supplementary Figure S6. Temozolomide increases gut permeability in aged mice. Serum values of FITC-Dextran were increased in aged mice that received Temozolomide compared to vehicle ($p<0.05$, $t=2.51$, $df=7$).

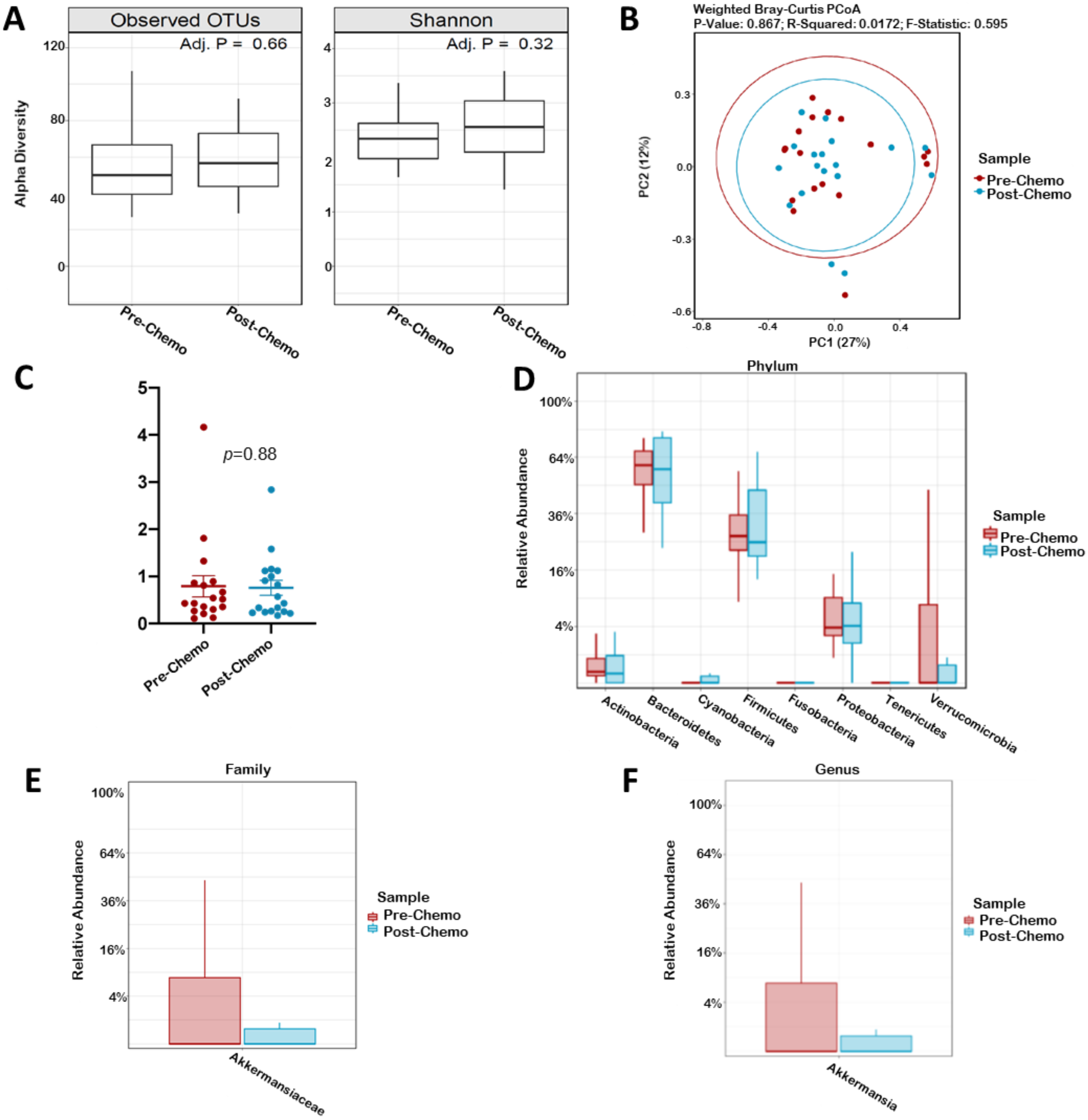

Supplementary Figure S7. A-F. Effect of Glioma and Temozolomide in human patients. Comparison of Pre-Chemo (2-4 weeks after surgery but prior to chemoradiation and Post-Chemo (after 6 weeks of chemoradiation) (n=18) **A**. Observed OTU in alpha diversity (*p*= 0.66) or the Shannon diversity (*p*= 0.32). **B.** Beta-diversity Weighted Bray-Curtis PCoA (*p*= 0.867, R-Squared 0.01729, F-statistic 0595). **C.** F/B ratio (two-tailed t test, p=0.9073, t = 0.1174 df=34). **D.** Relative abundance of Verrucomicrobia (*p*= 0.924) **E.** Family level relative abundance of *Akkermansiaceae* (*p*= 0.965) **F.** Genus level relative abundance *Akkermansia* (*p*= 1.00).

Supplementary Table 1. Demographic Characteristics of Controls (N=21).

Variable	N (%)
Age group	
18-54	10 (48)
> 55	6 (29)
N/A	5 (23)
Sex	
Male	11 (52)
Female	7 (33)
N/A	3 (15)
Race/ethnicity	
White/Caucasian	3 (15)
African American	0 (0)
Hispanic	2 (10)
Asian/Pacific Islander	4 (19)
N/A	12 (56)

N/A: Not available
